# Delay in Diagnosis of Cervical Cancer in Afghanistan: A Pilot Cross-Sectional Survey

**DOI:** 10.3389/frph.2021.783271

**Published:** 2021-12-09

**Authors:** Cecilia Acuti Martellucci, Mohammad Delsoz, Shohra Qaderi, Shekiba Madadi, Divya Bhandari, Akihiko Ozaki, Sayed Hamid Mousavi

**Affiliations:** ^1^Department of Medical Sciences, University of Ferrara, Ferrara, Italy; ^2^Kabul University of Medical Science, Kabul, Afghanistan; ^3^Medical Research Center, Kateb University, Kabul, Afghanistan; ^4^Student Research Committee, School of Medicine, Shahid Beheshti University of Medical Sciences, Tehran, Iran; ^5^Medical Governance Research Institute, Tokyo, Japan; ^6^Department of Breast Surgery, Jyoban Hospital of Tokiwa Foundation, Fukushima, Japan; ^7^Afghanistan National Charity Organization for Special Diseases (ANCOSD), Kabul, Afghanistan

**Keywords:** cervical cancer, Afghanistan, delay, diagnosis, poverty & inequality

## Abstract

**Objectives:** The present study aimed to investigate the potential delays in healthcare seeking and diagnosis of women with cervical cancer (CC) in Afghanistan.

**Methods:** Clinical records of three hospitals in Kabul were searched for CC cases, and the women identified were interviewed by a trained physician using a semi-structured questionnaire. The main outcomes were the prevalence of potential delays over 90 days (1) from symptoms onset to healthcare seeking (patient delay), and (2) from first healthcare visit to CC diagnosis (healthcare delay). Information was also collected on: type and stage of CC, diagnostic test utilized, familiarity for CC, signs and symptoms, treatment type, and potential reasons for delaying healthcare seeking.

**Results:** 31 women with CC were identified, however only 11 continued their treatment in the study hospitals or were reachable by telephone, and accepted the interview. The mean age was 51 ± 14 years, and only 18.2% had a previous history of seeking medical care. Patient delay was seen in 90.9% of the women (95% CI: 58.7–99.8), with a median of 304 ± 183 days. Instead, healthcare delay was found in 45.4% (95% CI: 16.7–76.6), with a median of 61 ± 152 days. The main reasons for patient delays were unawareness of the seriousness of the symptoms (70.0%) and unwillingness to consult a healthcare professional (30.0%). None of the women ever underwent cervical screening or heard of the HPV vaccination.

**Conclusions:** Given the global effort to provide quality health care to all CC patients, Afghanistan needs interventions to reduce the delays in the diagnosis of this cancer, for instance by improving all women's awareness of gynecological signs and symptoms.

## Introduction

Cervical cancer (CC), caused by oncogenic human papillomavirus (HPV) (1), was responsible for an estimated 604,000 new cases and 341,000 deaths in 2020 and is the leading cause of cancer death in 36 Low- or Middle-Income Countries (LMIC) (2). Cervical screening and HPV vaccination are powerful strategies for the prevention of CC, especially when combined (3). Such promise was the driving factor for the launch, in November 2020, of the Global Initiative to Eliminate Cervical Cancer, by the World Health Organization (WHO) (4).

Most High-Income Countries (HIC) have already deployed both screening and vaccination, with Japan renewing the effort to raise vaccine coverage (5). Instead, concerning LMIC, pilot projects for HPV vaccination are ongoing in some African countries (6, 7), and national or regional screening and vaccination programmes were started in the Pacific area (8), as well as in all countries of Central and South America, although with different degrees (9).

However, despite this unprecedented global momentum, countries which are unstable due to conflict are being left behind (6). This is the case, in particular, of Afghanistan, where cancer registry data dates back to 1969 (10), and the only recent study on cancer patients was performed on refugees in Pakistan (11, 12). In such a context where preventive interventions are lacking, the prompt diagnosis of CC is fundamental to improve prognosis, and yet diagnostic delays are common in LMIC (13, 14). The International Agency for Research on Cancer estimates an age-standardized CC incidence rate of 10.4 on 100,000 people in Afghanistan (15), and yet updated evidence is lacking on the management of women with CC, and on whether they receive a prompt diagnosis (16). Therefore, the aim of the present study was to analyze the characteristics of CC patients in Afghanistan, with a special focus on the potential delays in healthcare seeking and diagnosis.

## Methods

### Setting and Population

This was a cross-sectional study including women with a diagnosis of CC, in three hospitals: Afghanistan National Pathology Center, Shefajo Hospital, and Jamhuriat Hospital, all of which are located in Kabul. A fourth hospital, contacted by the researchers, did not provide access to their clinical files. Data was collected through hospital records screening and participants interviews. The latter were needed because the records were often incomplete, and rarely provided information on the potential delays in healthcare seeking and/or diagnosis which were the main object of this study. Therefore, consecutive CC diagnoses made between October 2020 and June 2021 were identified through paper clinical files, and interviews were carried out between March 2021 and June 2021 either face-to-face during a hospital visit, or by telephone, when the women's contact was available. Trained physicians collected the informed consent, interviewed the patients, and recorded the answers on an Excel spreadsheet. The sample size was estimated assuming a two-tailed alpha of 0.05, and 85% prevalence of delay from symptom onset to healthcare seeking >90 days (13). It was calculated that a sample of 25 women was needed to keep the 95% Confidence Intervals (CI) within ± 20%. The study was approved by the Kateb University Ethics Committee on February 21 2021, with code AF.KU.HREC.03.

### Variables

The semi-structured questionnaire used in this study was developed through an in-depth literature review and is available from the researchers upon request (13, 17, 18). Socio-demographic information was collected on: age, residence province, occupation, years of schooling, marital status, perceived economic status, number of children, contraceptive use, previous healthcare use, comorbidities, and medicine use. No further questions, for example on the women's knowledge of cervical cancer, were administered, in order to obtain the highest possible response rate to the questions on diagnosis delay, which were the main focus of the present study. For women who declared that they did not believe their gynecological symptoms required a clinician's consultation, “Unawareness of symptoms' seriousness” was reported as their reason for patient delay. Clinical information was collected on: type and stage of CC (19), diagnostic tests utilized, familiarity for CC, signs and symptoms, treatment type, and potential reasons for delaying healthcare seeking.

### Data Analysis

The primary outcomes were the prevalence (95% CI) of delays >90 days (1) from signs and symptoms onset to healthcare seeking (patient delay), and (2) from first healthcare visit to CC diagnosis (healthcare delay) (20). Importantly, the median (IQR) delay times were also calculated for the whole sample. Socio-demographic and clinical data are presented through summary statistics: mean ± standard deviation (SD) were used for normally distributed continuous variables, while median ± inter-quartile range (IQR) were used for non-normally distributed continuous variables. Finally, categorical ones were described through percentages. All analyses were done using Stata 15 (Stata Corp., College Station, TX, USA).

## Results

**Figure 1:**
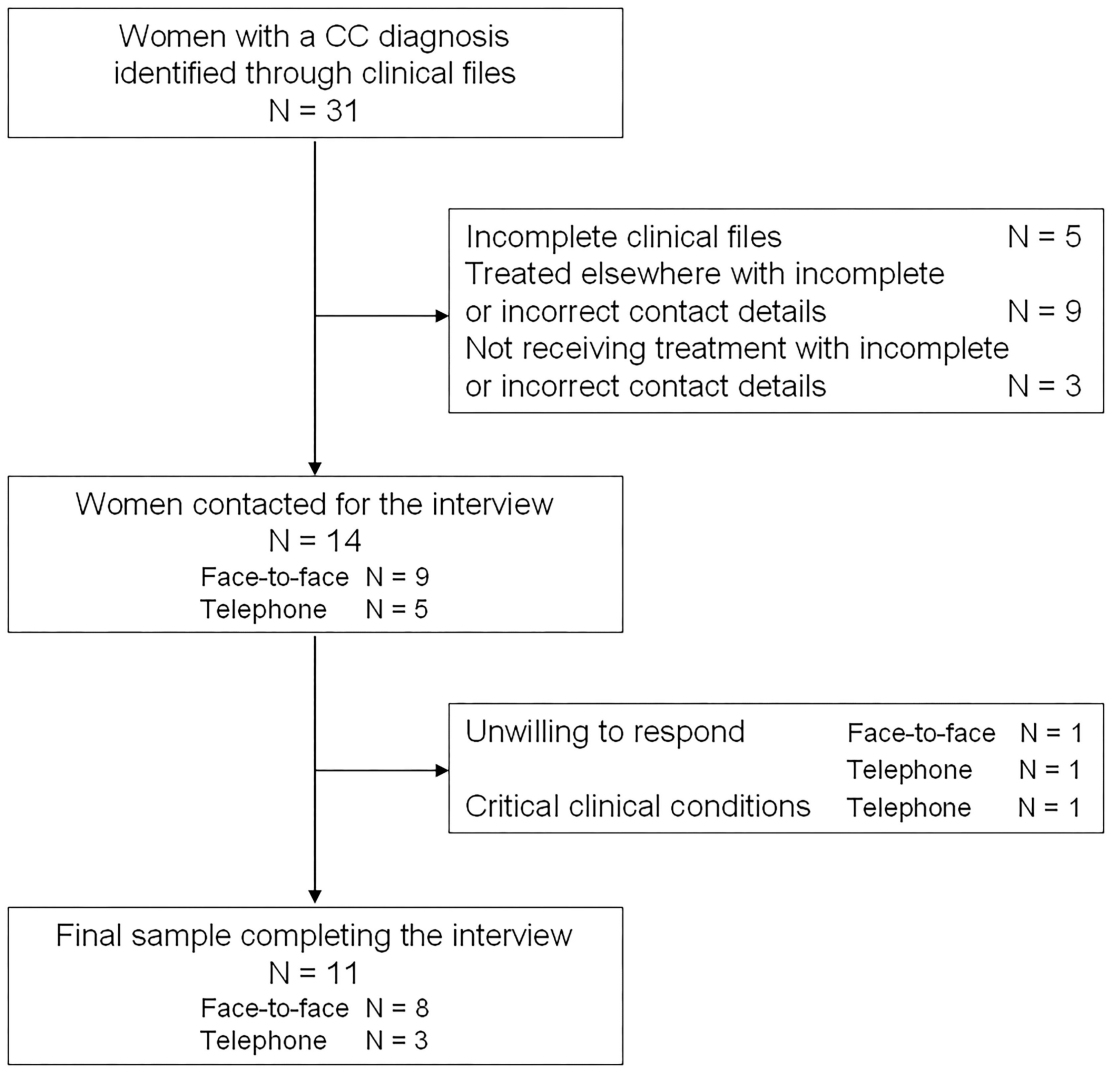
Flow-chart of study participation.

Clinical files for 31 women were retrieved in the three hospitals during the study period. Of these, we were able to contact only 14 women: nine which continued to receive treatment in the study hospitals, and five more whose contact details were available. One woman refused the face-to-face interview, and two women refused the telephone one, for a final sample of 11 women. Eight were interviewed in person and three by telephone (Figure 1).

In the study sample, the mean age was 51 ± 14 years, none of the women smoked tobacco, 63.6% of them were from Kabul, and 63.6.4% were married, while the rest were widowed. Almost all of them were housewives and had more than one child, up to a maximum of 11. Also, schooling years varied greatly between 0 and 12, and the women reported having either average or low economic status. More than half of the women reported no use of contraceptives in the previous 10 years, 27.3% reported using oral contraceptives, and 18.2% used condoms (Table 1).

**Table 1:** Characteristics of participants (*N* = 11).

**Women's characteristics**	Prevalence, ^%*^
Age, mean (SD)	51.1 (14.2)
≤45 years old	45.4
**Residence**	
Kabul	63.6
Other province	36.4
**Marital status**	
Married	63.6
Widow	36.4
**Number of children**	
1	9.1
2-6	72.7
7–11	18.2
**Schooling years**	
0	36.4
2–5	27.2
6–12	36.4
**Occupation**	
Housewife	90.9
Employed	9.1
**Perceived economic status**	
Poor	54.6
Average	45.4
**Contraceptives use in the last 10 years**	
None	54.5
Condom	18.2
Oral contraceptives	27.3

* *Percentages unless otherwise specified*.

Concerning the main outcomes, 90.9% of the women (95% CI: 58.7–99.8) had a patient delay >90 days, with a median of 304 ± 183 days while 45.4% (95% CI: 16.7–76.6) had a healthcare delay >90 days, with a median of 61 ± 152 days (Table 2). Time from symptoms onset to healthcare seeking ranged from 2 months to 4 years, and time from the first visit to the diagnosis ranged from one to 8 months. The main reasons for the patient delays were the lack of awareness regarding the severity of symptoms (70.0%) and the unwillingness to consult a healthcare professional (30.0%). This was consistent with the low proportion of women (18.2%) who reported previous use of healthcare, and with the absence of medication users in the sample (Table 2). Notably, the only woman who took <90 days from symptoms onset to seek care was misdiagnosed at her first visit and only received the CC diagnosis 6 months later.

**Table 2:** Outcomes and clinical characteristics of participants (*N* = 11).

**Women's characteristics**	Prevalence, %^*^
**Delays in days**	
Patient delay, median (IQR)	304 (183)
> 90 days, % (95% CI)	90.9 (58.7–99.8)
Healthcare delay, median (IQR)	61 (152)
> 90 days, % (95% CI)	45.4 (16.7–76.6)
**Clinical characteristics**	
**Histopathology**	
Squamous cell carcinoma	72.7
Adenocarcinoma	27.3
**Cancer staging** ^**†**^	
II	45.4
III	18.2
IV	9.1
Unavailable	18.2
**Signs and symptoms**	
Abnormal vaginal bleeding	90.9
Abnormal vaginal discharge	63.6
ower abdominal or back pain	63.6
Headache	45.4
Fatigue	36.4
Abdominal mass	18.2
Syncope	9.1
**CC diagnosis**	
Biopsy	63.6
Imaging	18.2
Colposcopy	18.2
**Treatment**	
Surgery (hysterectomy ± partial or total removal of annexes)	54.5
Surgery and chemotherapy	18.2
In planning at the time of data collection	27.3
Familiarity for cervical cancer	18.2
Comorbidities^‡^	18.2
Previous use of healthcare	18.2
**Reasons for patient delay** **(N** **=** **10)**	
Unawareness of symptoms' seriousness	70.0
Unwillingness to consult healthcare professionals	30.0
Difficulty accessing healthcare	10.0
Mistreated by healthcare professionals at first visit	10.0
Prioritizing pressing family matters	10.0
Visiting more >1 healthcare professional before diagnosis	63.6

* *Percentages unless otherwise specified. FIGO (International Federation of Gynecologists and Obstetrics) 2018 staging classification (19). Comorbidities included self-reported major cardiovascular, respiratory, and kidney diseases, and cancer: in the present sample only two women reported having hypertension. IQR, inter-quartile range. CI, confidence intervals*.

Squamous cells carcinomas and adenocarcinomas were noted among 72.7 and 27.3% of participants respectively. While 45.4% of the women had stage II cancer, 18.2% had stage III and 9.1% had stage IV, for 18.2% it was not possible to retrieve the staging. The most frequent signs were abnormal vaginal bleeding (90.9%) and discharge (63.6%), while common symptoms were lower abdominal or back pain (63.3%), headache (45.4%), and fatigue (36.4%—Table 2). Finally, none of the women ever underwent cervical screening or had heard of the HPV vaccination.

## Discussion

The present cross-sectional study, which interviewed women with cervical cancer treated by three hospitals in Kabul, Afghanistan, found widespread considerable delays in healthcare seeking after the onset of signs and symptoms, and fairly frequent, although shorter, delays in diagnosis after the first contact with a healthcare provider. Importantly, in all patients, the overall diagnostic delay from onset of symptoms to CC diagnosis was greater than the maximum 90 days threshold cited in the literature (17, 21). These findings suggest that women in Afghanistan likely have insufficient awareness about abnormal gynecological conditions, but also that healthcare facilities may have the capacity to provide them with prompt diagnosis and treatment for such conditions.

Available evidence on CC in LMIC suggests that diagnostic delays are common, and appear to be driven mostly by patient delays. Indeed, median patient and healthcare delays were respectively 53 and 11 days according to one study on 122 women in North-Eastern India (18), 80 and 36 days according to another study on 210 women in Southern India (14), and 70 and 55 days in the third study on 110 women in Nepal (22). The longest patient delay was found by one assessment of 410 women in Ethiopia, with a median of 120 days (13). One exception is Morocco, where a research on 190 women found median patient and healthcare delays of 6 and 48 days, respectively (23). In the present study we observed the longest patient delay (median 304 days) thus far, and also the longest healthcare delay (median 61 days). The striking patient delay, in particular, suggests that interventions are urgently needed to promote timely healthcare seeking.

Finally, one recent investigation of brought-in-dead women in Zambia suggested that many do not even seek care at all, as only one fifth of the deceased due to CC had received a diagnosis, a phenomenon which should also be investigated in Afghanistan through further studies (24). Indeed, the high loss to follow-up indicated a high frequency of treatment refusals in the present study.

While no previous research studied patient or healthcare delays in Afghan women with cervical cancer, the lack of timely cancer diagnosis for Afghan people has been reported in four previous papers (11, 12, 25, 26), two of which inevitably focused on more easily reachable refugee populations (12, 25). Indeed, it is an established practice for many Afghans to flee both the conflicts and the poor medical facilities, in order to seek care in neighboring countries like Pakistan. Coupled with the high costs of therapy (16), this could explain the very high loss to follow up (65%) in the present study's hospitals (11, 26). An investigation of cancer diagnoses at a hospital in Lahore, Pakistan, reported treating 68 CC patients from Afghanistan from 1995 to 2017, of which 34% had stages III or IV (12). This is similar to the proportion found in the present study and suggests that the observed delays in diagnosis may have been substantially unchanged in the last two decades.

In this study over 60% of the women had to consult multiple providers before reaching a diagnosis, notwithstanding the very poor quality of the retrieved clinical files. Similar findings are shown by studies from Ethiopia, India, Nepal, and even England, which found diagnostic delays attributable to healthcare providers in a proportion of women with CC ranging 20 to 84% (18, 20, 22). At the same time, however, the healthcare delay observed here was much smaller than the patient delay, suggesting that clinicians' assessment, although not ideal, is not the major driver of the overall delay. Additionally, a 2016 overview of the state of cancer care in Afghanistan even warned that CC mortality is comparatively higher than that of cancers of other sites, due mainly to inequities in healthcare access and ignorance about gynecological cancers (27). Indeed, while the healthcare delays depend heavily on the availability of personnel and resources (27), longer patient delay has been associated with lower literacy (and consequently lower awareness of cervical cancer) (13, 14, 17, 18), greater distance from healthcare facilities (14, 17, 28), older age (14, 28), lower economic status (13, 14), missing cervical screening (18), but also local cultural practices and fear of people knowing (21). These are substantially confirmed by the present findings, as the Afghan women in our sample also underestimated signs and symptoms and were unwilling to seek care.

In the framework of the aforementioned WHO Global Initiative to Eliminate Cervical Cancer, it was calculated that, with HPV vaccination, two lifetime screening tests, and timely treatment of pre-invasive and invasive disease, over 185,000 cervical cancer deaths could be averted in Afghanistan between 2020 and 2120, (3) and thus the cost-effectiveness of a vaccination program is being evaluated in the country (29). However, in 2021 Afghanistan is destabilized not only by the SARS-CoV-2 pandemic (30), but also by the return to power of the Taliban, which has previously been associated with poorer women's health status (31–34). Accordingly, humanitarian organizations working locally keep appealing to donors and policymakers to drastically increase the accessibility and affordability of quality healthcare (35). Nevertheless, the prevention and effective treatment of cervical cancer could also benefit from simpler, bottom-up actions, especially considering that patient delays seem much greater than healthcare ones in the country. Interventions should be developed to raise awareness among the women about which signs and symptoms to notice, and about the practice of screening, which could potentially save many lives.

### Strengths and Weaknesses

This is the first study investigating the extent of the delay in healthcare seeking and diagnosis of women with CC in Afghanistan, and their clinical conditions. Three different hospitals were involved, and the semi-structured questionnaire was rigorously administered by trained physicians in order to gain comprehensive information on the delays and the reasons behind them (36).

However, the present study also has limitations which must be considered. First, the small sample size increased greatly the uncertainty around the estimated proportions of women with delays in healthcare seeking and diagnosis. Non-etheless, even with the measured uncertainty, patient delay was observed for at least 59% of CC patients, and the overall low numbers of CC patients identified through clinical files are consistent with previous literature on cancer diagnoses in Kabul hospitals (37). Second, it was beyond the scope of the present study to investigate the factors beyond healthcare delays: the women reported the recommendations received from the clinicians, but the later could not be interviewed with regard to their workload or knowledge. Also, the self-reported nature of the great part of our data suggests that it is subject to social desirability bias, and therefore the women could have underreported their delay in healthcare seeking. Notably, during one telephone interview, the patient's sister took the phone and said that the patient had symptoms for longer than declared but hid them from the family. Should this be confirmed as a widespread tendency in future studies, the recommendation to raise awareness among the female population would become even more important.

## Conclusions

In the absence of cancer registries, and with very limited treatment possibilities, cervical cancer in Afghanistan is hard to investigate and control. The present study showed considerable delays in diagnosis, mainly attributable to lack of awareness of the disease and unwillingness to seek care even among symptomatic women. Due to the limited sample size, further investigation is urgently needed to confirm the alarming entity of the observed patient delay for CC, and also to assess possible predictors of a longer delay, in order to design more precise strategies to address this issue. Regardless, given the uncertain policy agenda of Afghanistan's new government, one advisable course of action is to promote the women's awareness of specific gynecological signs and symptoms. Concurrently, the lack of resources for adequate therapy also needs to be promptly addressed.

## Data Availability Statement

The original contributions presented in the study are included in the article/supplementary materials, further inquiries can be directed to the corresponding author/s.

## Author Contributions

SQ, MD, CA, and AO conceived and designed the study and wrote the manuscript. SM and DB helped collect data. CA performed the statistical analysis and wrote the initial draft of the manuscript. SHM confirmed the eligibility of the participants for the study. All authors approved the final version of the manuscript.

## Conflict of Interest

The authors declare that the research was conducted in the absence of any commercial or financial relationships that could be construed as a potential conflict of interest.

## Publisher's Note

All claims expressed in this article are solely those of the authors and do not necessarily represent those of their affiliated organizations, or those of the publisher, the editors and the reviewers. Any product that may be evaluated in this article, or claim that may be made by its manufacturer, is not guaranteed or endorsed by the publisher.
